# Contrast sensitivity isocontours of the central visual field

**DOI:** 10.1038/s41598-019-48026-2

**Published:** 2019-08-12

**Authors:** Agnes Y. J. Choi, Lisa Nivison-Smith, Jack Phu, Barbara Zangerl, Sieu K. Khuu, Bryan W. Jones, Rebecca L. Pfeiffer, Robert E. Marc, Michael Kalloniatis

**Affiliations:** 1 Centre for Eye Health, The University of New South Wales, Kensington, New South Wales Australia; 2 School of Optometry and Vision Science, The University of New South Wales, Kensington, New South Wales Australia; 3 Department of Ophthalmology, Moran Eye Center, University of Utah, Salt Lake City, Utah United States

**Keywords:** Biomarkers, Retinal diseases

## Abstract

Standard automated perimetry (SAP), the most common form of perimetry used in clinical practice, is associated with high test variability, impacting clinical decision making and efficiency. Contrast sensitivity isocontours (CSIs) may reduce test variability in SAP by identifying regions of the visual field with statistically similar patterns of change that can be analysed collectively and allow a point (disease)-to-CSI (normal) comparison in disease assessment as opposed to a point (disease)-to-point (normal) comparison. CSIs in the central visual field however have limited applicability as they have only been described using visual field test patterns with low, 6° spatial sampling. In this study, CSIs were determined within the central 20° visual field using the 10-2 test grid paradigm of the Humphrey Field Analyzer which has a high 2° sampling frequency. The number of CSIs detected in the central 20° visual field was greater than previously reported with low spatial sampling and stimulus size dependent: 6 CSIs for GI, 4 CSIs for GII and GIII, and 3 CSIs for GIV and GV. CSI number and distribution were preserved with age. Use of CSIs to assess visual function in age-related macular degeneration (AMD) found CSI guided analysis detected a significantly greater deviation in sensitivity of AMD eyes from normal compared to a standard clinical pointwise comparison (−1.40 ± 0.15 dB vs −0.96 ± 0.15 dB; p < 0.05). This work suggests detection of CSIs within the central 20° is dependent on sampling strategy and stimulus size and normative distribution limits of CSIs can indicate significant functional deficits in diseases affecting the central visual field such as AMD.

## Introduction

Perimetry is a common diagnostic test used for evaluating both central and peripheral visual function in the management of ocular and neurologic diseases^1^. Static perimetry involves placing stationary stimuli at discrete locations in the visual field. A contrast sensitivity value is then estimated at each location by adjusting the luminance to determine the minimum light increment required to detect the stimulus^1,2^. This generates a two-dimensional plot of sensitivity (conventionally reported as stimulus attenuation in dB) within a prescribed area of the visual field. Static perimetry differs from kinetic perimetry, in which a stimulus is typically moved along different meridians from the periphery towards fixation until the observer first detects it^1,2^, forming a contour of equal sensitivity in the visual field known as an isopter^3^ or kinetic isocontour.

The most commonly performed perimetry test protocol in clinical practice is white-on-white standard automated perimetry (SAP) using a static, Goldmann III stimulus. Results from this test are conventionally presented as single contrast sensitivity values for each location tested^4–6^. High test variability in SAP however leads to issues in clinical decision making (i.e. deciding whether an abnormal sensitivity value is due to disease or test variability) and clinical efficiency (due to the need for retesting) using this technique^7–9^.

Recent studies suggest test variability in static perimetry may be improved through the use of contrast sensitivity isocontours (CSIs)^10–13^. CSIs are regions of the visual field assessed by static perimetry that have statistically similar changes in contrast sensitivity with age or other parameters, analogous to isocontours in kinetic perimetry^10,11^. CSIs allow locations in a static perimetry test grid to be analysed collectively as a group with other statistically similar locations instead of individual locations. This may reduce test variability by increasing the number of samples assessed for sensitivity for each region of the visual field^11–13^. CSIs may also improve assessment of ocular disease by moving beyond one-to-one comparisons of each location in the visual field and allowing abnormal test locations to be compared with multiple other locations belonging to the same CSI. CSI organisation has also been shown to correspond with anatomical distribution of ganglion cells^14^ suggesting that they may be able to contribute to more accurate structure-function models^11,15^.

So far, a maximum of four CSIs have been identified in the central 20° visual field from analysis of the 30-2 visual field paradigm of the Humphrey Field Analyzer (HFA)^11^. This paradigm tests 77 locations across the central 60° visual field but only 16 test locations fall within the central 20° due to its coarse 6° degree sampling strategy. Previous work indicates large sensitivity changes up to 0.9 log units (with Goldmann size I stimulus) occur across the central 20°^16^. The transition between complete and partial spatial summation for Goldmann size I and II stimulus also occurs at approximately 6° and 10° from the fovea respectively^17^. Thus establishing CSIs in this region with only 16 test locations may not indicate the true scope of CSIs in the central visual field.

The 10-2 test paradigm of the HFA tests 69 locations across the central 20**°** visual field using a 2° sampling strategy. This paradigm with high sampling frequency may be more suitable for determining CSIs in the central visual field. Improving the diagnostic power of the 10-2 visual field test is also highly relevant to clinical practice as accurate assessment of diseases involving the central visual field is essential as they generally have devastating consequences on vision and quality of life^18,19^. Assessing the central visual field with tests of low sampling frequency has also been reported to miss/underestimate glaucomatous damage of the macula^20–22^. Thus, the aim of this study was to describe CSIs in the central 20° visual field based on the 10-2 test paradigm. CSIs were identified using pattern recognition^11^, a method which objectively analyses image datasets by detecting patterns unique to groups of objects with or without prior training^23^. This new data was applied to a group of eyes with early age-related macular degeneration (AMD) to determine if CSIs could assist in assessing visual function in a disease affecting central vision and associated with significant variability in function within the central 20° in the early stages of disease^24^.

## Methods

### Participants

Ethics approval for this study was given by the University of New South Wales Ethics Committee and the study conducted in accordance with the tenets of the Declaration of Helsinki. All participants gave written informed consent prior to the study.

Fifty-six participants with no history of visual abnormalities were recruited based on previous work indicating ideal sample size for normative visual field populations^25^. Thirty-seven participants had their average contrast sensitivity measurements reported in a previous study^17^. Participants underwent a standard eye examination at the Centre for Eye Health (CFEH) at the University of New South Wales, including retinal photography (Kowa Non-Mydriatic Nonmyd WX3D) and optical coherence tomography (Macular Cube 512 A-scans x 128 horizontal scan lines and Optic Disc Cube 200 A-scans x 200 horizontal scan lines; Cirrus OCT, Carl Zeiss Meditec) to confirm no detectable ocular pathology that would affect visual field results. Participants with lenticular or corneal opacities, signs or symptoms of central retina abnormalities or other co-morbidities which may affect central visual field were excluded. Twenty-three participants with early to intermediate AMD (based on Ferris *et al*.^26^) were also recruited and subjected to similar testing and exclusion criteria (lenticular or corneal opacities and co-morbidities only) as normal participants. AREDS gradings^27^ for these participants were 1 (2 participants), 2 (14 participants), 3 (3 participants) and 4 (4 participants). Table 1 outlines the characteristics of each cohort.

**Table 1:** Characteristics of participants for this study.

	Normal	AMD
Demographics		
Number of participants	56	23
Sex		
Male	23	15
Female	33	8
Age (years)		
Mean ± SD	43 ± 14	70 ± 6
Range	20–67	56–80
Ethnicity		
Caucasian	26 (46.4%)	23 (100%)
Asian	28 (50%)	—
Mixed	2 (3.6%)	—
Ocular parameters		
Best corrected visual acuity (BCVA)		
Median	20/16	20/20^−1^
Range	20/25^−1^ to 20/12.5^−1^	20/25 to 20/12.5^−2^
Refractive error (Rx)		
Median	−0.13	0.75
Range	+2.50 to −6.75 (one subject with Rx = −8.25)	−1.00 to +4.25

Refractive error is given as spherical equivalent in diopters.

### Visual field testing

Sensitivities (in dB) of all participants were measured using the 10-2 Full Threshold paradigm of the Humphrey Field Analyzer (background luminance: 10 cd/m^2^ stimulus duration: 200 ms)^28^. The Full Threshold strategy was selected over other strategies such as the SITA Standard strategy as it does not rely on prior knowledge for threshold determination or utilise proprietary post-processing steps^29,30^ This ensured thresholds at a given location were obtained once a predetermined statistical level of testing certainty is reached and not influenced by any prior models or assumptions^4,28^. Each participant was tested twice in a single eye for stimulus sizes GI to GV (subtending 0.1°, 0.21°, 0.43°, 0.86°, and 1.72°, respectively) with test order randomised. The sensitivity measurements for each test location were averaged. AMD participants were tested in a single eye for GIII only. All tests were performed with natural pupils, with any required refractive correction placed in the HFA trial frame, and the short term fluctuation option enabled (this option allows for two threshold values to be noted at locations in the 10-2 visual field to indicate test reliability). Participants were given adequate breaks to minimise test fatigue. For all participants, tests were repeated if the reliability criteria were below those specified by the manufacturer at the time of the study (greater than 33% false positives, 33% false negatives and 20% fixation losses). For normal participants, tests were also repeated if test-retest variability (based on the mean range of difference of sensitivity measurements at all locations) was greater than 3 dB based on an expected test variability of approximately 2.13 dB within the central 20° visual field reported by Heijl *et al*.^8^. For analysis, all data were converted to a right eye orientation. This conversion has been widely used by our group and others^8,11,16,17,31^.

### Organisation of sensitivity measurements

The D’Agostino & Pearson normality test (α = 0.01) was performed to confirm that sensitivity measurements from all normal participants for all 69 test locations follow a normal distribution. Only one non-central test location (6° inferior, 4° nasal of the fovea) did not follow normality with a single stimulus size (GV). Subsequently, the ROUT method (coefficient Q = 10%) was used to remove outliers of dB values at each test location from the dataset^32^. This method removes sensitivity values that do not meaningfully contribute to the distribution limits without drastically affecting the central tendency statistics and ensures points that fall outside the normative distribution due to organic loss are more likely to be identified^25^. No outlier removal was performed on the AMD dataset.

Normal participant data were organised in two ways before pattern recognition analysis – age-based and age-corrected groups. For the age-based group analysis, participant data were separated into five decade age groups: 20–29 (n = 13, mean = 26 ± 3 years), 30–39 (n = 13, mean = 33 ± 3), 40–49 (n = 10, mean = 45 ± 3), 50–59 (n = 10, mean = 56 ± 3), and ≥ 60 (n = 10, mean = 63 ± 2). For the age-corrected analysis, sensitivity measurements of all 56 participants were corrected to 50 year-old equivalent as previously described^8^ then participant data randomly divided into 6 groups (4 groups of 9 and 2 groups of 10). Several studies have employed age-correction methods to enable pooling of sensitivity data^11,12,16,17,29,31,33,34^.

### Pattern recognition analysis

**Figure 1:**
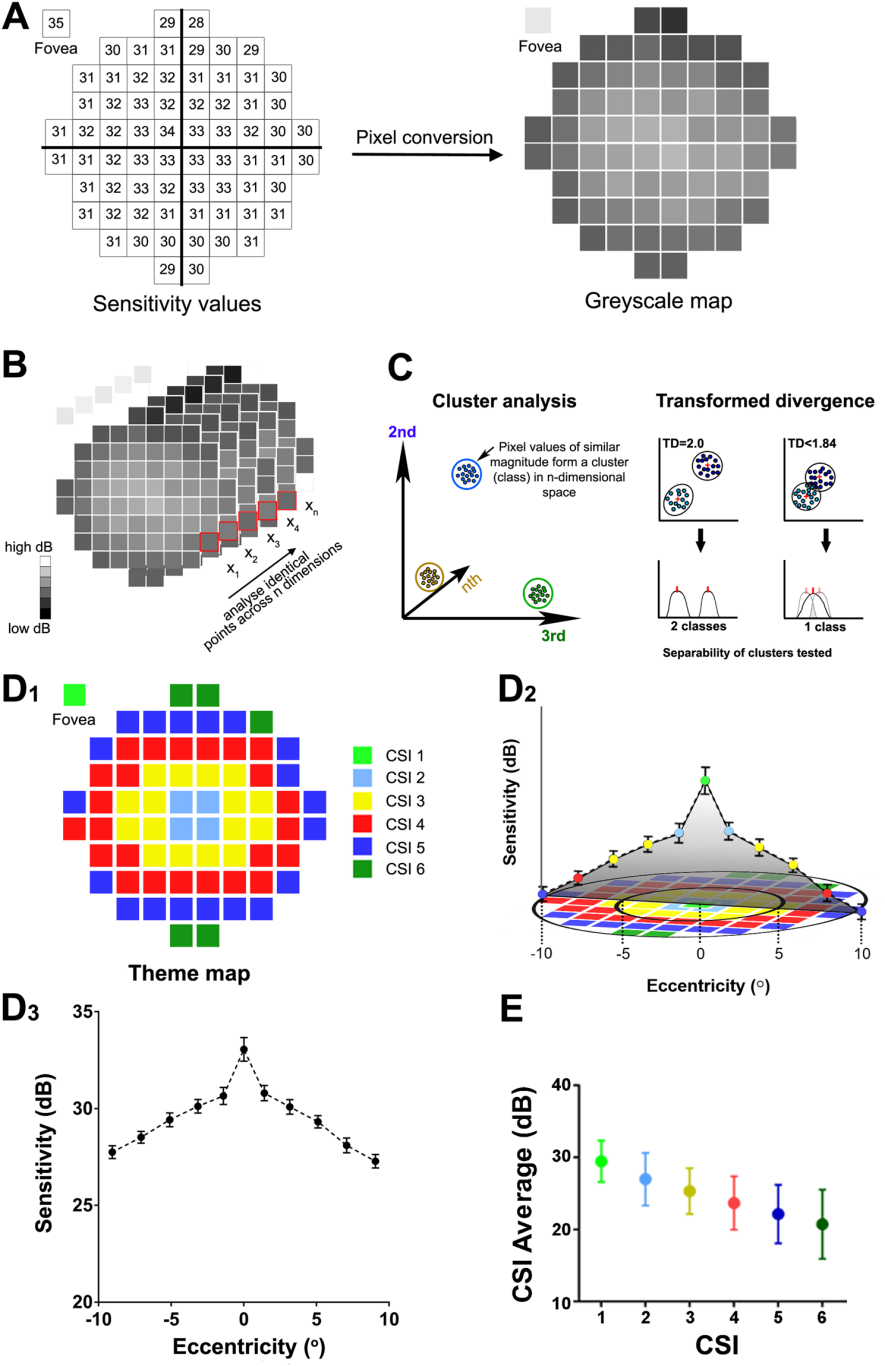
Schematic of CSI detection using pattern recognition. (**A**) Sensitivity values (dB) were converted to pixel values between 0–255 to generate greyscale maps. (**B**) Greyscale maps were arranged in stacks with a stack representing a particular group of data (age group stack shown as an example). Test locations with similar sensitivity values were clustered into classes in n-dimensional space and separability of classes (where class means are represented by red crosses/ticks) was determined based on transformed divergence (D_T_) statistic (**C**). A D_T_ value of >1.84 was used to indicate two separable classes (indicating correct separation of >95%). Below this value, classes were merged. (**D**_**1**_) Classes were color coded to generate a theme map showing locations in the visual field that have statistically similar sensitivity values (i.e. CSIs). (**D**_**2**_) The relationship between the CSIs and a cross-section of the Hill of Vision along the horizontal meridian is illustrated in a stylized image. (**D**_**3**_) The Hill of Vision plot along the horizontal meridian demonstrated peak sensitivity at the fovea and decreasing sensitivity with increasing eccentricity from the fovea. (**E**) Mean sensitivity values of CSIs were determined by finding the average sensitivity across all test locations within a CSI (i.e. CSI deconvolution) to generate dot plots of mean CSI contrast sensitivity as a function of age.

Sensitivity measurements (expressed in dB) were converted to pixel values (Fig. 1A) ranging from 0–255 by multiplying of the output by 5.25 to maximize the range of pixel values with ‘0’ corresponding to the lowest dB value (represented as black) and ‘255’ corresponding to a highest dB value (represented as white). The pixel range of 0–255 was used as it is a common range used by image processing equipment^35^ and represents 256 possible grey levels (i.e. 8 bits per pixel or an 8-bit image). The use of different multiplication factors does not affect the final classification result^11,14^. Pixel values were then used to generate greyscale maps of the 10-2 visual field (Fig. 1A) using Adobe Photoshop CS5 Extended (v12.1 × 64, Adobe Systems Incorporated). Greyscale maps were imported into the pattern recognition software (Geomatica, PCI, Canada) and arranged into stacks with each stack representing a particular group of data (e.g. a decade age group in the age-based analysis, or a random group in the age-corrected analysis; Fig. 1B). Pattern recognition was used to investigate the n-dimensional dataset (n = 5 in the age-based analysis, n = 6 in the age-corrected analysis). Stacks were analysed by the ISODATA clustering algorithm^36^ where pixel values of similar magnitude formed clusters in multidimensional space called theme classes (Fig. 1C; left)^37^. Separability of theme classes was subsequently assessed based on the transformed divergence (D_T_) value (Fig. 1C; right) where a D_T_ value of 0 indicates complete overlap of the theme classes (i.e. probability error in classification (p_e_) = 1) and a D_T_ value of 2 indicate complete separation between two classes (i.e. p_e_ = 0)^38,39^. In this study, a criterion of D_T_ >1.84 was defined as statistically separable, which translates to an approximate probability of correct classification of >95%^40,41^. In addition, as in our previous work, CSIs were subjected to an additional clinical criterion whereby the mean sensitivity of a CSI needed to be at least 1 dB different to the adjacent CSI as this is the minimum sensitivity resolution reported for the HFA (i.e. a 1 dB step was selected as this is the minimum step size difference reported by the HFA)^11^. A theme map was generated by color coding each test location in the 10-2 grid based on its theme class depicting each CSI (Fig. 1D_1_) and the relationship between the CSIs and a cross-section of the Hill of Vision is depicted in a stylized image (Fig. 1D_2_) with the Hill of Vision plot along the horizontal meridian being shown separately in Fig. 1D_3_. We deconvoluted the data by taking the sensitivity values of all locations which compose the CSIs and generating dot plots of mean sensitivity and distribution for each CSI (Fig. 1E).

### Hierarchical cluster analysis

To confirm that sample size did not influence CSI generation, cluster analysis was performed on simulated large data sets. Simulated datasets of n = 50 and 5000 visual fields for GIII were generated by random sampling of a single visual field from the existing age-corrected normal cohort data with replacement for ‘n’ times. Simulated dataset of n = 200 visual fields for GIII was generated by random sampling of six visual fields from the existing age-corrected normal cohort data with replacement for 200 times. Cluster analysis was then performed using hierarchical cluster analysis with SPSS Statistics Version 22.0 (IBM Corporation, New York, NY, USA). Hierarchical cluster analysis was used in place of pattern recognition due to limitations in the latter in regards to the number of input channels (i.e. 16 channels in the ISODATA unsupervised clustering). Pairs of clusters were merged if d’ < 1, starting from the lowest d’ value and stopping until all pairs had d’ > 1, where d’ is calculated by the formula: d’ = |(χ_1_ − χ_2_)|/(0.5 × (σ_1_^2^ + σ_2_^2^)^0.5^), where χ_1_ and χ_2_ and σ_1_ and σ_2_ are the means and standard deviations of clusters 1 and 2 respectively (see Phu *et al*.^11^).

Hierarchical cluster analysis was also performed to confirm that the scale used for pixel conversion did not have an effect on CSI generation. In order to do this, existing sensitivity data in dB (i.e. logarithmic scale) was converted to 1/Lambert (i.e. linear scale) and hierarchical cluster analysis was performed on the converted sensitivity data.

### Sampling strategy comparison

To determine the effect of sampling strategy on CSI detection, pattern recognition was repeated for two additional datasets composed of (1) 10-2 test grid paradigm with an additional 12 paracentral test locations extracted from the 30-2 test grid paradigm and (2) 30-2 test grid paradigm but only including locations within the central 20° visual field. Contrast sensitivity for locations in the 30-2 test grid paradigm were extracted from Phu *et al*. (n = 60 normal participants)^11^ and were already corrected to a 50 year-old equivalent. Approximately 50% of the participants within the dataset extracted from Phu *et al*.^11^ (i.e. 29 participants) also participated in this study. Pattern recognition and subsequent analysis were performed as described above.

### Bootstrapping

Bootstrapping was performed to determine the mean sensitivity and lower distribution limit (5^th^ and 1^st^ percentile) of the CSIs from age-corrected analyses. Briefly, CSIs were resampled, whereby a subset of the sensitivity values (of size *x*, where *x* is the size of the total sample) was randomly extracted from the original cohort of 56 participants (i.e. a resample) with replacement (such that each sample could potentially be extracted more than once). This resampling process was repeated 200 times to obtain bootstrapped descriptive statistics for each stimulus size (see Phu *et al*.^25^, for a detailed description of the bootstrapping procedure). This provides a comparison for parametric versus non-parametric (distribution free) limits.

### Analysis of sensitivity measurements in AMD using CSIs

Sensitivity measurements from AMD participants were age-corrected to a 50 year-old equivalent as described for normal participants and then compared in a pointwise fashion to: (1) normative data for that specific test location and stimulus size (i.e. standard clinical pointwise analysis), (2) normative distribution limits for the CSI that the test location was assigned to (i.e. CSI guided analysis) or (3) normative distribution limits for relevant CSIs determined from bootstrapping. Test locations in AMD eyes were flagged as outside normal limits if the sensitivity was below the 5^th^ percentile of the normative distribution limits.

### Statistical analysis

Statistical analysis was performed using GraphPad Prism (v7, GraphPad Software, Inc., La Jolla, CA, USA). Chi-squared analysis was used to confirm similarities in sex and BCVA between decade age groups. A two-way ANOVA was performed to determine how age and eccentricity affect contrast sensitivity measurements (in dB) for GI to GV while a one-way ANOVA was used to assess eccentricity effect on contrast sensitivity measurements for GI to GV for age-corrected data.

## Results

### CSI detection through age-based analysis

**Figure 2:**
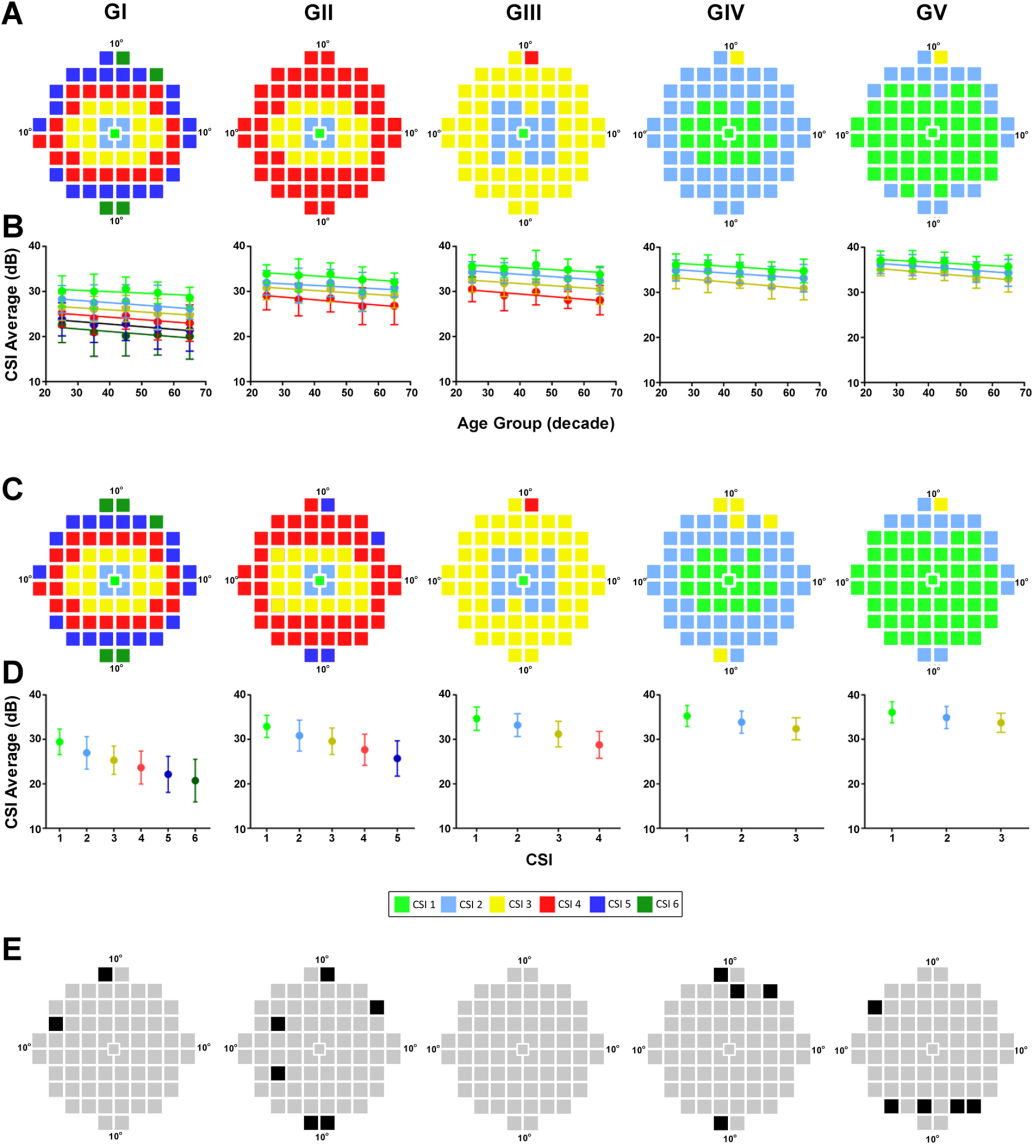
CSIs detected in the central 20° visual field. (**A**) Theme maps indicating age-based CSIs in the central visual field for GI-GV and (**B**) resulting dot plots showing mean sensitivity for CSIs as a function of age. (**C**) Theme maps of age-corrected CSIs following conversion of all data to a 50 year-old equivalent and (**D**) resulting dot plots showing mean sensitivity for different CSIs. (**E**) Difference maps highlighting test locations assigned to different CSIs in age-based versus age-corrected analysis (black). Note, pseudocolor CSI maps are specific for each analysis so that CSIs are not color-coded the same across stimulus sizes (e.g. the dB value of a test location shaded yellow in GI is different to that of a test location shaded yellow in GIII). All data points represent the mean ± two standard deviations.

Pattern recognition analysis revealed CSIs within the central visual field based on change in contrast sensitivity as a function of age (Fig. 2B). Separability was at a maximum between most CSIs (i.e. D_T_ value = 2.0) with slightly lower values between adjacent CSIs in the periphery indicating that the central classification was ≥95% correct (Supplementary Table 1). CSIs were dependent on stimulus size with a greater number of CSIs being found for small stimuli: 6 CSIs for GI, 4 CSIs for GII and GIII (although CSI 4 for GIII consists of only one isolated point), and 3 CSIs for GIV and GV. Between stimulus sizes GI and GII, decrease in the number of CSIs resulted from test locations in the peripheral 10-2 test grid paradigm becoming less separable (i.e. all 25 test locations in CSI 5 and CSI 6 in GI formed part of CSI 4 in GII). For larger stimulus sizes such as GIII, GIV and GV, test locations in central CSIs became less separable (i.e. CSI 1, 2 and 3 for GIV was composed of 28%, 71% and 1% of test locations respectively whilst for GV, CSI 1, 2 and 3 was composed of 71%, 28% and 1% of test locations respectively).

The mean contrast sensitivity values of each CSI (based on the average of all test locations allocated to that CSI) as a function of age are shown in Fig. 2B. For all stimulus sizes, mean CSI contrast sensitivity decreased with eccentricity (two-way ANOVA; GI - GV: *P* < 0.0001) and decreased with age (GI - GV: *P* < 0.0001). The range of mean CSI sensitivities however differed between stimulus sizes with the average difference between the most central CSI (i.e. CSI 1) and most peripheral CSI (i.e. CSI 6) for GI being 8.98 dB across all ages versus 2.48 dB for CSI 1 to CSI 3 for GV.

### CSI detection through age-corrected analysis

All contrast sensitivity data were corrected to 50 year-old equivalent sensitivities (Supplementary Figure 1) and reanalyzed to determine if changes due to eccentricity alone could allow CSI generation (or if age was a necessary contributing factor). Pattern recognition analysis identified 6 CSIs for GI, 4 CSIs for GII and GIII and 3 CSIs for GIV and GV (Fig. 2C). When compared to age-based analysis, the number and location of these age-independent CSIs were mostly similar suggesting age was not a major factor in CSI generation (Fig. 2E). CSIs consisting of a single location such as CSI 4 in GIII and or CSI 3 in GV also remained in the age-corrected analysis confirming these locations were separate CSIs.

Mean CSI contrast sensitivity decreased with eccentricity for all stimulus sizes (one-way ANOVA: GI - GV: *P* < 0.0001). The range of sensitivities between CSIs was also notably reduced with increasing stimulus size: for GI, the difference between the mean of the most central CSI 1 to the most peripheral CSI 6 was 8.75 dB whilst the range for GV for CSI 1 to CSI 3 was only 2.36 dB (Fig. 2D).

### Validation of CSI map generation

**Figure 3:**
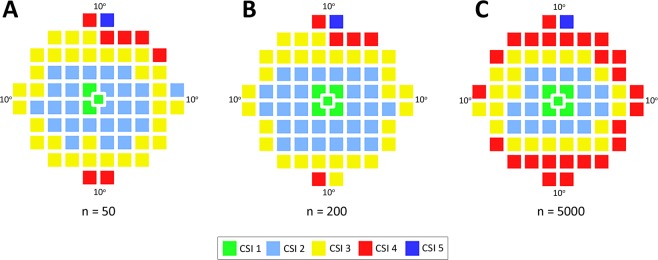
CSIs detected with varying sample size. Theme maps indicating CSIs in the central visual field for GIII based on hierarchical cluster analysis of simulated visual field data of (**A**) n = 50, (**B**) n = 200 and (**C**) n = 5000.

We subsequently validated that the generation of CSI maps was not influenced by external factors such as sample size or choice of scale for pixel conversion. For sample size, CSI map generation was repeated using hierarchical cluster analysis and a simulated dataset of 50 age-corrected visual fields (to reflect the sample size of the current study) and larger sample sizes of 200 and 5000 visual fields using the GIII stimulus. This analysis was based on the hypothesis that if sample size influenced the number of clusters generated, a large sample size (such as 5000) would result in each visual field location representing a single cluster. Figure 3 demonstrates however this was not the case and for all sample sizes tested, cluster analysis identified groups of visual field locations with statistically similar mean sensitivity (i.e. CSIs). Although there was some discordance between the exact locations assigned to each CSI, the same number of CSIs and overall concentric distribution was evident for the different sample sizes.

To confirm that the scale used for pixel conversion did not influence CSI distribution, cluster analysis was performed following data transformation of contrast sensitivity from dB to 1/Lambert (linear scale) and compared with that performed with contrast sensitivity in dB. Conversion resulted in an overall similar distribution of CSIs although there were some differences in the peripheral CSIs (Supplementary Figure 2). These results suggest that the general concentric distribution of CSIs is maintained following conversion of sensitivities from logarithmic (dB) to linear (1/Lambert) scale and supports the concept that scaling the data does not influence pattern recognition outcomes.

### CSI detection incorporating additional test points within the central 20**°**

**Figure 4:**
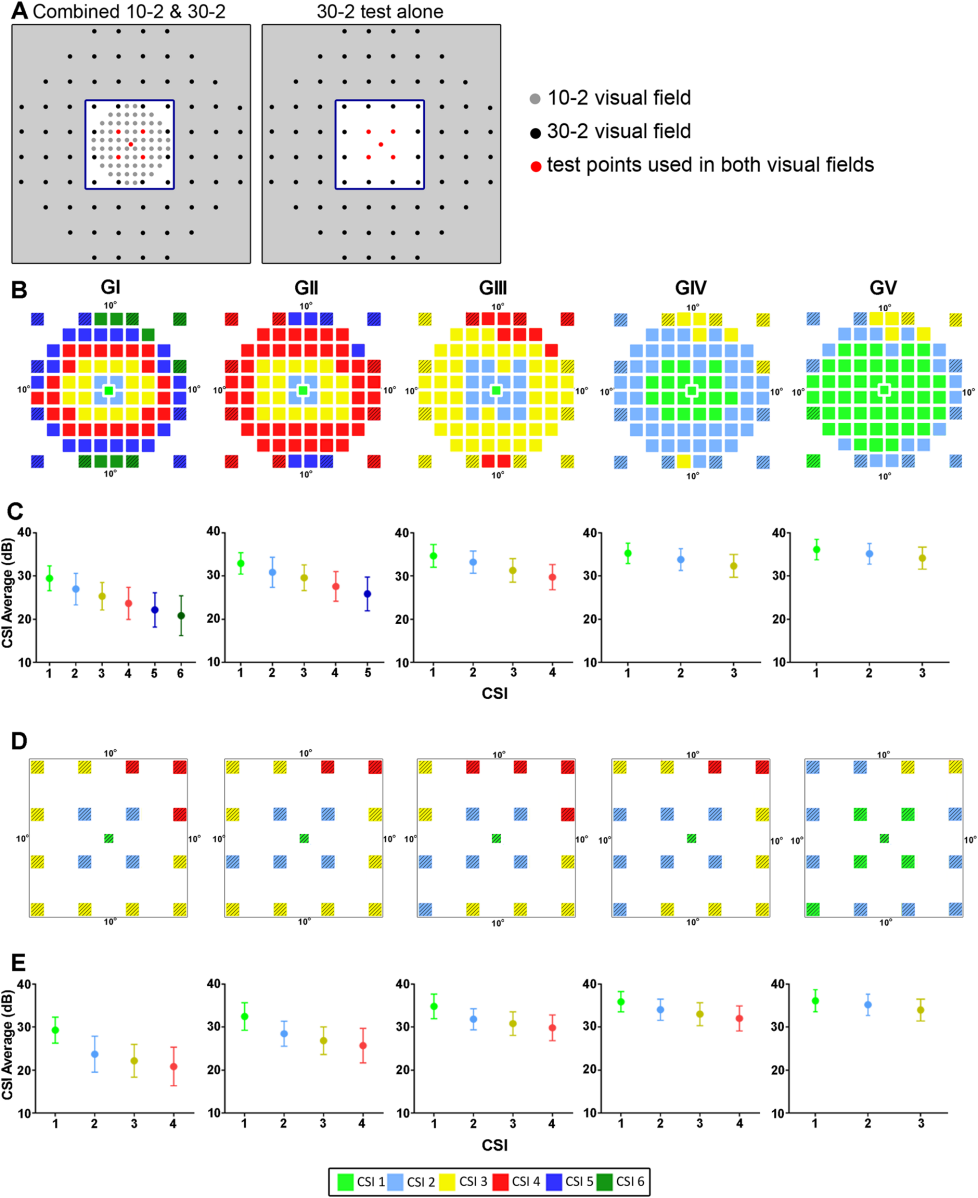
CSIs detected with inclusion of central 30-2 test grid paradigm locations. (**A**) Two alternate test grids to assess the central 20° visual field: the 10-2 test grid paradigm with an additional 12 paracentral test points of the 30-2 test grid paradigm and 17 tests points of the 30-2 test grid paradigm within the central 20° alone. Blue box delineates the central 20° visual field. (**B**) Theme maps of CSIs and (**C**) resulting dot plots of mean sensitivity as a function of CSI for the combined 10-2 and 30-2 test grid paradigm (striped) and (**D**) theme maps of CSIs and (**E**) dot plots for 30-2 test grid paradigm alone. Pseudocolor maps are specific for each analysis and data points in dot plots represent the mean ± two standard deviations.

Although the 10-2 test grid paradigm has 69 test locations within the central 20° visual field, only 8 of these locations are at the 10° border. The 30-2 test grid paradigm has 12 test locations at this eccentricity thus to ensure the maximum number of CSIs had been identified in the central 20° visual field, we performed pattern recognition of the 10-2 test grid paradigm with the inclusion of 12 paracentral test points of the 30-2 test grid paradigm (Fig. 4A). Age corrected contrast sensitivities for these 12 additional test points were extracted from Phu *et al*.^11^ and Fig. 4B shows the resulting theme maps of the combined dataset. Additional test points of 30-2 test grid paradigm were classified into the peripheral CSIs resulting in no change in the number of CSIs in the central 20°. For CSIs that consist of a single location such as CSI 4 in the GIII (red, Fig. 2C), nearby paracentral points from the 30-2 test grid paradigm were also classified into this CSI. Thus CSIs consisting of a single location appeared to be a result of the limits of the test locations within the 10-2 test grid paradigm. The mean contrast sensitivity of CSIs generated with additional test points were not significantly different to those generated with test points from the 10-2 test grid paradigm alone for GI, GII and GIV (two-way ANOVA; p = 0.27–0.93). A significant difference was observed for GIII (p < 0.01) and GV (p < 0.001) with post hoc analysis indicating a significant difference in mean CSI contrast sensitivity occurring for the peripheral CSIs.

### CSI detection in the central 20**°** using low sampling strategy

Although a limited number of CSIs were detected for the central 20° visual field using the 30-2 test grid paradigm^11^, this analysis included all locations within the 30-2 test grid paradigm (both in and outside 20°). Thus we reassessed the 30-2 test grid paradigm using only the 17 tests locations within the central 20° to confirm a reduced number of CSIs are detected with a low sampling strategy. Our analysis found 4 CSIs were detected for GI to GIV and 3 CSIs for GV with CSIs less dependent on stimulus size (Fig. 4D). CSIs closer to the centre of the test grid still demonstrated a significant increase in contrast sensitivity values (one-way ANOVA: GI - GV: *P* < 0.0001; Fig. 4E).

### Normative distribution limits of CSIs

**Figure 5:**
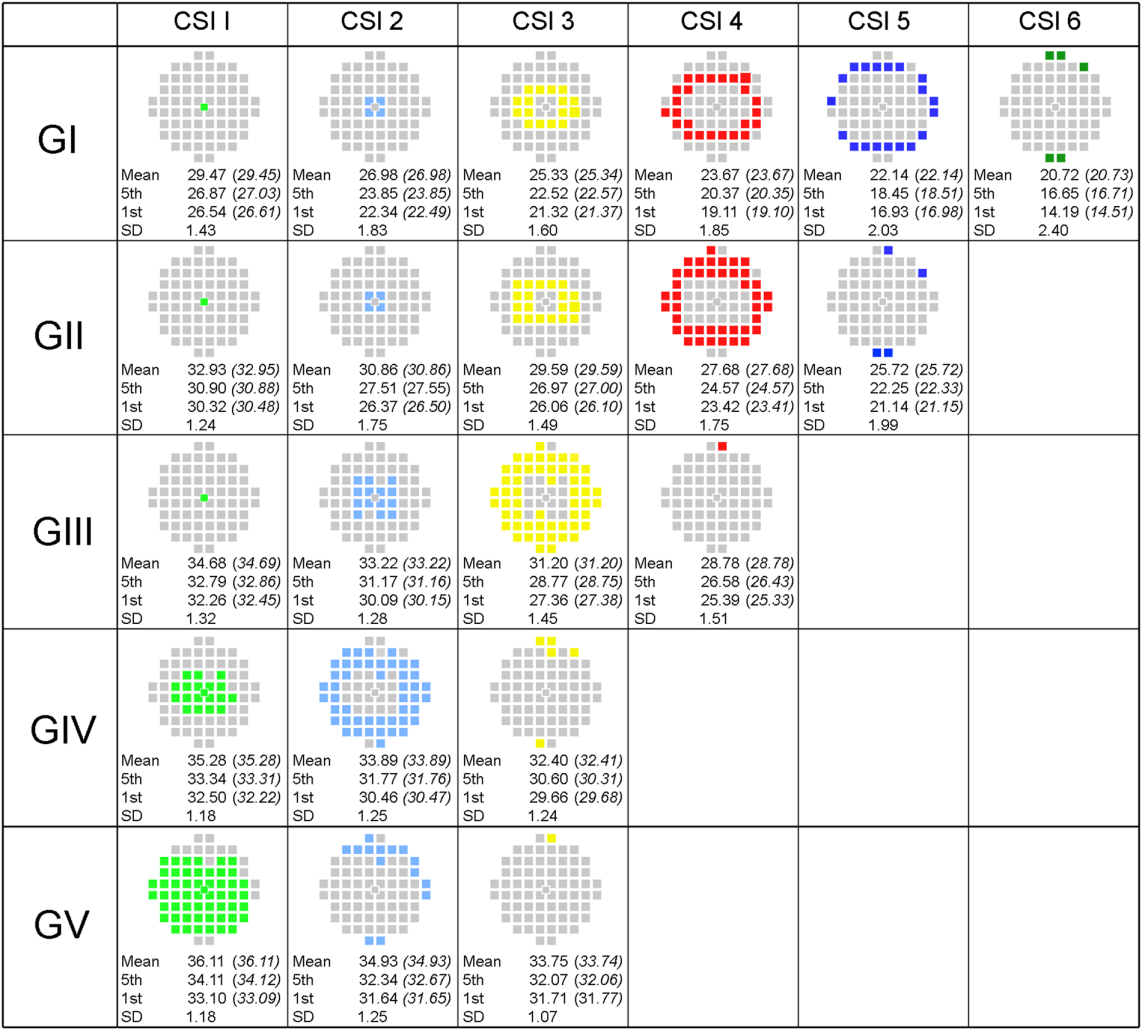
Mean contrast sensitivity and distribution limits (5th and 1st percentiles) for each CSI. Limits were determined for GI-V for the 50 year-old equivalent sensitivities for each CSI (color coded in the test grid). Contrast sensitivity values were calculated from the sample cohort (n = 56) or following non-parametric bootstrapping of sample data with 200 times of resampling (italic). Standard deviation values for GI-GV for each CSI were also determined for the age-corrected sample cohort.

As age and the addition of extra paracentral points from the 30-2 visual field did not alter the number of CSIs in the central 20°, the normal distribution limits for age-corrected CSIs was determined to allow easy application to clinical practice. The mean sensitivity, standard deviation and lower distribution limits (5^th^ and 1^st^ percentiles) for each CSI for each stimulus size was determined for the age-corrected dataset (Fig. 5). A bootstrapped dataset where data was assessed following 200 times of resampling was obtained and its summary statistics are provided in Fig. 5 (full bootstrapped dataset not shown). Little difference was found between either dataset for the mean or lower limits of sensitivity.

### Application of CSIs to assess visual function in AMD

**Figure 6:**
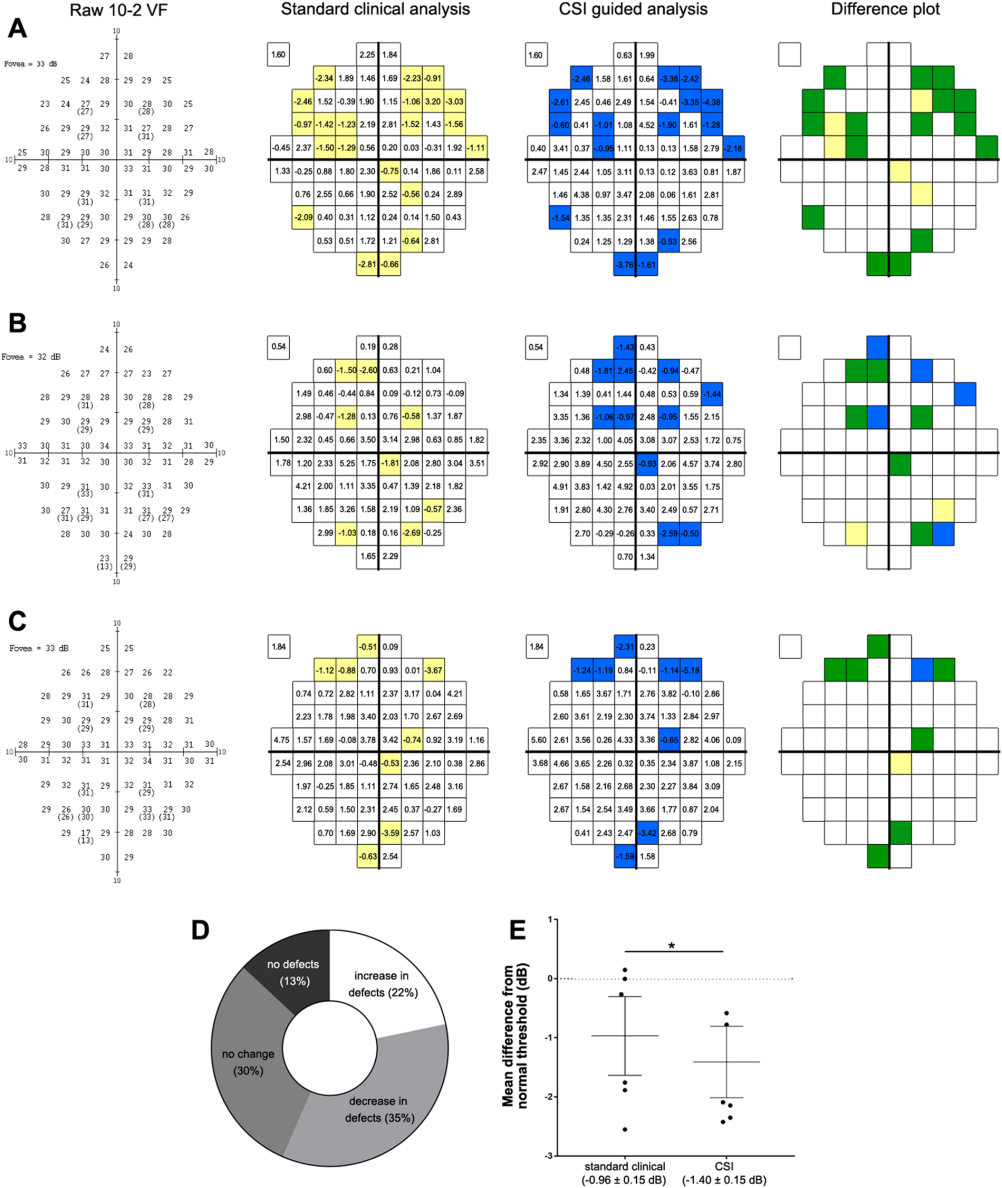
Analysis of central visual function of AMD eyes using standard clinical pointwise analysis and CSI guided analysis. Examples from eyes of a (**A**) 73 year-old Caucasian female, (**B**) 72 year-old Caucasian male and (**C**) 77 year-old Caucasian male with intermediate AMD tested using the standard GIII stimulus and analysed in a pointwise fashion using normative distribution limits of individual test locations for GIII (standard clinical pointwise analysis) or normative distribution limits of CSIs for GIII (CSI guided analysis). Difference plots indicate defects missed by CSI guided analysis (yellow), new defects identified by CSI guided analysis (blue) or defects flagged by both analyses (green). Comparison between standard clinical pointwise analysis and CSI guided analysis for all AMD eyes (n = 23) of the number of flagged locations (i.e. defects) (**D**) and mean difference (± standard deviation) from sensitivity of normal eyes (**E**). *=p < 0.05.

As a proof of concept, CSIs were used to assess function in the central visual field in a cohort of early to intermediate AMD participants. Examples of three AMD eyes used for analysis are shown in Fig. 6. For standard clinical pointwise analysis where contrast sensitivity of the AMD eyes was age-corrected and assessed against the mean, age-corrected sensitivity of the normal population (Supplementary Figure 1) at each test location for GIII, locations in the AMD eyes were flagged if the contrast sensitivity at that location was below the 5^th^ percentile of the normal population distribution (Fig. 6A–C, yellow squares). For CSI guided analysis where age-corrected sensitivity of each location for the AMD eyes were compared to the normative distribution limits of the CSI to which that location was assigned to (see Fig. 5), locations in the AMD eyes were flagged if the contrast sensitivity at that location was outside the normal distribution limits (Fig. 6A–C, blue squares). Locations that were flagged by both analyses were indicated on the difference plot (Fig. 6A–C, green squares). Overall, of all twenty-three AMD eyes, 22% demonstrated an increase in the number of locations flagged as outside normal limits using CSI guided analysis versus standard clinical pointwise analysis, 35% demonstrated a decrease and 30% demonstrated no change (Fig. 6D). Three eyes (13%) demonstrated no visual field locations with sensitivities below the 5^th^ percentile via either analysis (Fig. 6D).

A total of 92 locations in 23 AMD eyes were flagged as outside normal distribution limits using standard clinical pointwise analysis whilst only 85 locations were flagged using CSI guided analysis. Of the 62 locations flagged by both analyses however, CSI guided analysis demonstrated a significantly greater difference in sensitivity between AMD and normal eyes compared to standard clinical pointwise analysis (p < 0.05; Fig. 6E). Similar results were obtained when using limits derived through bootstrapping (data not shown). As a bootstrapped database consisted of a large collection of random samples derived from the original dataset of the normal participants, similarity in findings suggests no systematic issues within our normative database.

## Discussion

This study describes CSIs in the central visual field acquired using pattern recognition analysis of the 10-2 test grid paradigm. Four to six CSIs were identified in the central visual field, dependent on stimulus size. This was greater than the number of CSIs detected from analysis of the 30-2 test grid paradigm indicating a sampling strategy greater than 6° is necessary when assessing CSIs within this region. CSI number and distribution were maintained regardless of sample size and relatively unaffected by the addition of more paracentral points to the 10-2 test grid paradigm, suggesting a 2° sampling strategy may be sufficient to detect the maximum number of CSIs within the central 20° visual field. Analysis of visual function in AMD eyes guided by CSIs found a greater sensitivity loss compared to standard clinical pointwise analysis for locations outside normal distribution limits.

### Arrangement and number of CSIs appears dependent on the Hill of Vision

When CSIs of the central visual field were deconvoluted, we found that mean sensitivity for CSIs was eccentricity dependent, decreasing towards the periphery. This arrangement suggests CSIs predictably follow the shape of the Hill of Vision which is known to have the highest sensitivity at fixation and a gradual decline towards the periphery^17,42–45^. Further validating this hypothesis, a previous study establishing CSIs across the central 60° visual field showed a similar arrangement and distribution^11^. Anatomical correlates including retinal ganglion cells (RGC) and cone photoreceptors also show a similar center-periphery decline in cell density^46,47^. Yoshioka *et al*.^14^ and Tong *et al*.^15^ also demonstrated age-related RGC thickness loss in the macula as measured by OCT followed a concentric pattern similar to the CSIs observed in this study.

The Hill of Vision also explains the total number of CSIs detected in the central visual field being stimulus size dependent. Choi *et al*.^17^ previously demonstrated a steeper Hill of Vision using the HFA for smaller stimulus sizes with the average change in sensitivity from the fovea to the periphery of 9 dB for GI compared to 2 dB for GV in the central 20° visual field. This means small stimulus sizes provide a greater range of sensitivities over which to detect patterns of change using pattern recognition analysis, resulting in more statistically separable CSIs. This is reflective in CSI mean sensitivity which decreases with eccentricity for all ages and stimulus sizes but is less apparent for large stimulus sizes.

We also demonstrated that CSI distribution was not an artefact of sample size with similar CSI patterns for GIII following analysis of large simulated datasets. Our sample size analysis is consistent with Phu *et al*.^25^, who found a sample size beyond 60 normal participants for full-threshold visual field results provides distribution limits similar to that of 500 normal participants and the addition of more participants do not provide further information. Additionally, CSI generation was not an artefact of sensitivity scale and is consistent with our previous work which demonstrates scale does not affect cluster analysis in other visual field test grids or anatomical clusters determined from age-based assessment of GCL thicknesses^11,14,15^.

### CSIs can be generated independent of age

Several studies including the SITA-standard algorithm of the HFA^29^ have employed age-correction methods to enable pooling of sensitivity data of participants of different ages into a single range of normalized sensitivity values^11,12,16,17,29,31,33,34^. In this study, we also age-corrected to determine if CSI generation was dependent on age-related change. CSI number and location was similar between age-grouped and age-corrected data suggesting these CSI attributes are preserved with age. Thus regardless of age, the same locations contribute to CSIs and can be assessed collectively in the central 20° visual field for all individuals. The mean sensitivity of each CSI however decreased with increasing age indicating whilst the locations can be assessed together, the values at these locations have to be corrected for age or assessed against an age-matched population. Phu *et al*.^11^ reported that the rate of age-related loss in sensitivity was slower when using larger stimulus sizes compared with smaller sizes. We however found no significant difference in age-related rates of sensitivity decline across all stimulus sizes. This discrepancy may be attributed to the 30-2 test grid paradigm including more peripheral locations. Indeed we found slight, but statistically significant decreases in CSI mean sensitivity for CSIs consisting of a single location for GIII and GV when paracentral points from the 30-2 test grid paradigm were included in the CSI. We also found slight differences in CSI distribution between CSIs identified in the central 20° of the 30-2 test grid paradigm when all locations were analysed^11^ versus analysis of the central 17 tests points of the 30-2 test grid paradigm alone.

### CSIs in the 10-2 visual field can be useful in assessing central visual function in disease

SAP is associated with high test variability making comparisons at single test location problematic. Indeed, age-related changes in contrast sensitivity assessed through pointwise analysis has been shown to be highly variable^8,48^. CSIs can improve the diagnostic power of visual field tests by allowing multiple locations with identical characteristics to be averaged and analysed collectively. This has been demonstrated for glaucoma where detection of glaucomatous VFs was improved using a CSI-derived clustering map compared with the commercially available Glaucoma Hemifield Test (GHT)^13^.

In this study, we assessed the utility of CSIs for assessing visual function in eyes with early to intermediate AMD. Reduced visual function in early AMD has been reported using both static and flicker perimetry^49–52^ however, sensitive methods for defect detection in the central visual field are needed as the early stages of AMD are associated with significant variability in function within the central 6°^24^. We found CSI guided analysis resulted in fewer locations flagged as outside normal limits compared to standard clinical pointwise analysis. Interestingly, these ‘missed’ locations by CSI guided analysis often appeared to be isolated from other flagged locations and therefore may have been false positives. This notion is supported by the greater deviation in threshold from normal for locations flagged outside normal limits by CSI guided analysis compared to standard clinical pointwise analysis. This difference in the mean difference from normal threshold between the two analyses does not readily translate to the presence of bias in the CSI guided analysis. In fact, this difference may possibly be attributed to higher variability when locations are assessed individually (i.e. in standard clinical pointwise analysis) compared to when locations are assessed as a group (i.e. in CSI guided analysis). Furthermore, the fact that the absolute number of locations flagged by CSI guided analysis was the same or less than standard clinical pointwise analysis contradicts the possibility of bias in the CSI guided analysis. Considering there is a number of modified visual field paradigms demonstrating the ability to detect more defects and greater sensitivity reduction over standard clinical SAP, the addition of CSI guided analysis may further enhance these paradigms by reducing the false positive rate.

### Limitations

Age and disease-related factors such as poor fixation or fatigue were more likely to affect the AMD cohort than the normal cohort and lead to variability in contrast sensitivity measurements. To manage this, we implemented strict reliability criteria in visual field testing for both populations and limited testing within our AMD cohort to GIII only. Phu *et al*.^11^ demonstrated no clinically significant difference between contrast sensitivities of participants aged 45–55 years old and participants with sensitivities corrected to a 50 year-old equivalent suggesting variability associated with age is limited. The AMD eyes assessed in this study also had a minimum BCVA of 20/20 and thus no significant vision loss existed in our disease population.

Issues relating the potential combination of independent inter-subject variability and dependent intra-subject variability across locations in our normal cohort were also considered. We attempted to minimise (not eliminate) inter-subject variability by using a sample size which according to Phu *et al*.^25^ should be less significantly affected by inter-subject variability when generating a normative full threshold visual field database. It should be noted that although normative database limits stabilized when such a sample size was used, these limits quantify the level of, but do not remove inter-subject variability.

On the issue of intra-subject variability, there is evidence to indicate locations within the visual field are not actually independent but are significantly correlated^53^ and therefore have a measureable covariance. The fact that we found similar results between pointwise comparison of AMD participants with the normative and bootstrapped databases suggests no systematic variance within our normative database. The following points also suggest no systematic variation that could account for our findings: (1) we used the Full Threshold algorithm that makes no prior assumptions for threshold determination and thus thresholds were as independently determined as possible on the HFA device; (2) the large number of test points (69 points including the foveal point in the 10-2 test grid paradigm) maximizes spatial uncertainty^54^ suggesting that thresholds were independently determined in our study and other published studies^11,16,33^; and (3) the clustering algorithm used in our study (i.e. unsupervised partitioning of data into CSIs) does not take into consideration any spatial or location specific information thus the generation of CSIs was independent of any spatial input.

Although we described CSIs for all five Goldmann stimulus sizes available on the HFA, this study only demonstrated its utility in a disease cohort for the GIII stimulus size. The GIII stimulus is the most commonly used stimulus size in clinical practice and therefore this study confirms that CSIs could have immediate utility within current clinical practice protocols. Our previous work indicates GI and GII stimulus sizes operate within complete spatial summation in the central 20° visual field and could be more useful in the assessment of this region and therefore future work should assess the utility of CSIs with the other stimulus sizes. We also did not perform longitudinal evaluation and so it is not possible to determine whether the locations flagged as outside normal limits by either analysis are clinically significant or if the apparent increase in specificity conferred by CSI guided analysis may be accompanied by changes in sensitivity. Future studies comparing structure-function concordance of locations flagged outside normal limits through CSI guided analysis versus standard clinical pointwise analysis would be useful to further explore this concept.

## Conclusion

This study described CSIs within the central 20° using pattern recognition of the 10-2 visual field paradigm. Smaller stimulus sizes and higher sampling strategy led to a greater number of detectable CSIs. CSIs guided analysis detected visual field locations that were outside of normal limits in AMD eyes with greater defect depth than standard clinical pointwise analysis.

## Supplementary information

Supplementary material

## Data Availability

The datasets generated during and/or analysed during the current study are available from the corresponding author on reasonable request.
