# Supplementary material for: Contrast sensitivity isocontours of the central visual field
Source: Sci Rep. 2019 Aug 12;9:11603. doi: 10.1038/s41598-019-48026-2 (PMC6691009; doi:10.1038/s41598-019-48026-2)
Supplement: Supplementary file 1 — Supplementary material [file 41598_2019_48026_MOESM1_ESM.pdf]

Contrast sensitivity isocontours of the central visual field

Agnes Y.J. Choi[1,2], Lisa Nivison-Smith[1,2], Jack Phu[1,2], Barbara Zangerl[1], Sieu K. Khuu[2],

Bryan W. Jones[3], Rebecca L. Pfeiffer[3], Robert E. Marc[3], and Michael Kalloniatis[1,2]*

[1] Centre for Eye Health, The University of New South Wales, Kensington, New South Wales, Australia
[2] School of Optometry and Vision Science, The University of New South Wales, Kensington, New South Wales, Australia
[3] Department of Ophthalmology, Moran Eye Center, University of Utah, Salt Lake City, Utah, United States

*** Corresponding Author**
Michael Kalloniatis
Centre for Eye Health, UNSW Australia, Sydney, 2052, NSW, Australia.
Phone: Int +61 2 81150710
Fax: Int +61 2 81150799
Email: m.kalloniatis@unsw.edu.au

Supplementary Table 1: D_T for Pairwise Comparisons of CSIs across GI–V using pattern recognition analysis. D_T values indicating all CSIs are classified correctly to a level of 95% (D_T > 1.84), with most at 100% correct classification level (D_T = 2.00).

Age-group analysis	CSI1	CSI 2	CSI 3	CSI 4	CSI 5
**GI**					
2	2.00				
3	2.00	2.00			
4	2.00	2.00	1.92		
5	2.00	2.00	2.00	1.85	
6	2.00	2.00	2.00	2.00	2.00
**GII**					
2	2.00				
3	2.00	2.00			
4	2.00	2.00	1.92		
5	2.00	2.00	2.00	1.85	
6	2.00	2.00	2.00	2.00	2.00
**GIII**					
2	2.00				
3	2.00	1.86			
4	2.00	2.00	2.00		
**GIV**					
2	1.83				
3	2.00	2.00			
**GV**					
2	1.99				
3	2.00	2.00			

50 year old equivalent analysis	CSI 1	CSI 2	CSI 3	CSI 4	CSI 5
**GI**					
2	2.00				
3	2.00	2.00			
4	2.00	2.00	1.92		
5	2.00	2.00	2.00	1.85	
6	2.00	2.00	2.00	2.00	2.00
**GII**					
2	2.00				
3	2.00	2.00			
4	2.00	2.00	1.92		
5	2.00	2.00	2.00	1.85	
6	2.00	2.00	2.00	2.00	2.00
**GIII**					
2	2.00				
3	2.00	1.86			
4	2.00	2.00	2.00		
**GIV**					
2	1.83				
3	2.00	2.00			
**GV**					
2	1.99				
3	2.00	2.00			

GI

				21.2 ± 2.2	20.1 ± 2.1				
		22.1 ± 2.1	22.1 ± 2.1	22.4 ± 2.3	21.6 ± 2.0	21.7 ± 2.2	20.8 ± 2.9		
	22.3 ± 2.0	23.4 ± 1.7	23.8 ± 1.9	24.3 ± 1.7	23.6 ± 1.8	23.3 ± 1.8	22.9 ± 1.8	21.8 ± 2.3	
	23.1 ± 1.8	24.4 ± 1.7	25.8 ± 1.4	25.2 ± 1.4	25.3 ± 1.5	25.5 ± 1.3	24.1 ± 1.9	22.7 ± 2.2	
22.8 ± 1.8	23.8 ± 1.6	24.8 ± 1.6	25.9 ± 1.4	27.1 ± 1.8	27.0 ± 1.7	25.6 ± 1.5	24.7 ± 1.5	23.3 ± 2.2	21.6 ± 2.1
23.0± 1.7	24.1 ± 1.8	24.9 ± 1.6	25.6 ± 1.8	26.8 ± 1.9	27.1 ± 1.9	25.7 ± 1.7	24.7 ± 1.5	23.7 ± 2.1	22.1 ± 2.1
	23.1 ± 2.0	24.4 ± 1.8	25.5 ± 1.4	24.9 ± 1.7	25.2 ± 1.7	25.7 ± 1.7	24.2 ± 1.9	23.1 ± 1.8	
	22.6 ± 1.6	23.6 ± 1.9	23.9 ± 1.7	23.9 ± 1.7	23.8 ± 1.7	23.8 ± 1.6	23.3 ± 1.8	22.5 ± 1.9	
		21.9 ± 2.2	22.3 ± 1.6	21.8 ± 2.0	22.0 ± 1.9	22.5 ± 1.8	21.7 ± 1.9		
				20.5 ± 2.4	21.0 ± 2.1				

Fovea = 29.5 ± 1.4

GII

				26.2 ± 1.8	25.1 ± 1.9				
		26.9 ± 1.6	27.0 ± 1.7	27.3 ± 1.7	26.5 ± 1.7	26.6 ± 1.7	26.8 ± 1.7		
	27.4 ± 1.5	27.8 ± 1.7	28.2 ± 1.6	28.7 ± 1.5	28.3 ± 1.7	28.0 ± 1.7	27.8 ± 1.7	26.2 ± 2.0	
	27.9 ± 1.7	29.0 ± 1.3	29.8 ± 1.2	29.5 ± 1.4	29.3 ± 1.7	29.7 ± 1.2	28.5 ± 1.2	27.8 ± 1.3	
27.8 ± 1.4	28.5 ± 1.4	29.3 ± 1.6	30.2 ± 1.3	30.7 ± 1.8	31.0 ± 1.7	30.0 ± 1.5	29.2 ± 1.5	27.9 ± 1.7	27.0 ± 1.9
27.8 ± 1.6	28.5 ± 1.5	29.5 ± 1.4	30.1 ± 1.6	30.9 ± 1.7	30.9 ± 1.8	30.1 ± 1.6	29.3 ± 1.5	28.3 ± 1.7	27.4 ± 1.5
	28.2 ± 1.7	28.9 ± 1.5	29.9 ± 1.3	29.5 ± 1.5	29.8 ± 1.6	30.1 ± 1.3	29.0 ± 1.3	28.0 ± 1.5	
	27.0 ± 1.7	28.3 ± 1.7	28.5 ± 1.6	28.6 ± 1.9	28.6 ± 1.5	28.5 ± 1.6	28.3 ± 1.6	27.3 ± 1.6	
		27.0 ± 1.6	27.4 ± 1.7	26.8 ± 1.7	27.0 ± 1.9	27.4 ± 1.8	27.0 ± 1.8		
				25.6 ± 2.1	25.9 ± 1.9				

Fovea = 32.9 ± 1.2

GIII

				29.7 ± 1.3	28.8 ± 1.5				
		30.4 ± 1.3	30.5 ± 1.2	30.6 ± 1.2	29.8 ± 1.2	30.0 ± 1.3	29.7 ± 1.3		
	30.8 ± 1.3	31.3 ± 1.2	31.7 ± 1.3	31.4 ± 1.3	31.2 ± 1.3	31.4 ± 1.2	31.0 ± 1.4	30.1 ± 1.5	
	31.3 ± 1.3	32.1 ± 0.9	33.0 ± 1.2	32.5 ± 1.4	32.4 ± 1.2	32.9 ± 1.2	31.5 ± 1.4	31.1 ± 1.4	
31.0 ± 0.9	31.8 ± 1.3	32.5 ± 1.2	33.1 ± 1.2	33.9 ± 1.3	33.8 ± 1.4	33.0 ± 1.0	32.3 ± 1.2	31.3 ± 1.2	30.5 ± 1.5
31.4 ± 1.1	31.8± 0.8	32.4 ± 1.4	33.0 ± 1.2	33.9 ± 1.3	33.9 ± 1.2	33.1 ± 1.3	32.4 ± 1.0	31.6 ± 1.4	30.7 ± 1.5
	31.5 ± 1.2	32.3 ± 1.2	33.2 ± 1.1	32.4 ± 1.3	32.5 ± 1.3	33.2 ± 0.9	31.9 ± 1.1	31.0 ± 1.5	
	31.1 ± 1.1	31.6 ± 1.0	32.0 ± 1.1	31.7 ± 1.1	31.9 ± 1.1	31.8 ± 1.1	31.5 ± 1.0	30.8 ± 1.2	
		30.8 ± 1.4	30.9 ± 0.9	30.5 ± 1.3	30.8 ± 1.4	30.9 ± 1.3	30.5 ± 1.2		
				29.8 ± 1.3	30.2 ± 1.3				

Fovea = 34.7 ± 1.3

GIV

				32.5 ± 1.2	31.8 ± 1.3				
		33.1 ± 1.2	33.1 ± 1.0	33.3 ± 1.2	33.4 ± 1.3	32.9 ± 1.0	32.7 ± 1.2		
	33.5 ± 1.2	34.0 ± 1.1	34.0 ± 1.2	34.1 ± 1.3	33.7 ± 1.4	33.9 ± 1.1	33.7 ± 1.3	33.0 ± 1.2	
	34.1 ± 1.2	34.6 ± 0.9	35.3 ± 1.1	34.9 ± 1.2	34.6 ± 1.3	35.2 ± 1.2	34.4 ± 1.1	33.7 ± 1.1	
33.8 ± 1.2	34.5 ± 1.0	35.0 ± 1.0	35.4 ± 1.3	35.5 ± 1.2	35.4 ± 0.9	35.4 ± 0.8	34.7± 1.4	33.9 ± 1.1	33.2 ± 1.2
33.9 ± 1.1	34.6 ± 0.9	35.1 ± 1.0	35.6 ± 1.1	35.1 ± 1.2	35.3 ± 1.4	35.5 ± 1.3	35.8 ± 1.2	34.1 ± 1.1	33.4 ± 1.3
	34.2 ± 1.0	34.7 ± 1.2	35.5 ± 1.0	35.0 ± 1.1	34.9 ± 1.4	35.5 ± 1.1	34.6 ± 1.0	33.9 ± 1.3	
	33.7 ± 1.1	34.3 ± 0.7	34.3 ± 1.0	34.4 ± 1.0	34.5 ± 1.5	34.5 ± 1.1	34.3 ± 1.0	33.8 ± 1.1	
		33.4 ± 1.0	33.7 ± 0.8	33.3 ± 1.3	33.7 ± 1.3	33.5 ± 1.2	33.6 ± 1.2		
				32.7 ± 1.1	32.9 ± 1.2				

Fovea = 35.8 ± 1.4

GV

				34.4 ± 1.2	33.8 ± 1.1				
		35.0 ± 1.2	35.0 ± 1.0	35.3 ± 1.0	34.4 ± 1.3	34.8 ± 1.3	34.4 ± 1.4		
	35.5 ± 1.0	35.7 ± 1.1	35.8 ± 0.9	35.9 ± 1.3	35.3 ± 1.4	35.6 ± 1.1	35.6 ± 1.1	35.0 ± 1.1	
	35.9 ± 1.2	36.3 ± 1.1	36.8 ± 1.1	36.1 ± 1.1	36.2 ± 1.2	36.9 ± 1.0	36.0 ± 1.0	35.3 ± 1.2	
35.8 ± 1.1	36.3 ± 1.0	36.4 ± 1.1	36.6 ± 0.9	36.2 ± 1.4	36.2 ± 1.2	36.4 ± 1.2	36.3 ± 1.1	35.6 ± 1.3	35.3 ± 1.3
35.9± 1.1	36.2 ± 0.7	36.5 ± 1.1	36.8 ± 1.2	36.0 ± 1.2	36.2 ± 1.4	36.7 ± 1.2	36.5 ± 0.9	35.9 ± 1.1	35.1 ± 1.2
	36.0 ± 1.0	36.4 ± 1.0	37.1 ± 0.9	36.3 ± 1.1	36.4 ± 1.3	37.3 ± 1.2	36.3 ± 1.1	35.7 ± 1.4	
	35.7 ± 1.0	36.1 ± 1.0	36.3 ± 1.1	35.9 ± 1.0	36.2 ± 1.1	36.2 ± 1.0	36.0 ± 1.0	35.5 ± 1.2	
		35.5 ± 1.0	35.6 ± 1.2	35.5 ± 0.8	35.6 ± 1.2	35.4 ± 1.1	35.4 ± 1.0		
				34.9 ± 1.1	34.9 ± 1.1				

Fovea = 36.5 ± 1.6

GIII
(5th percentile)

				27.13	26.58				
		28.63	28.44	28.90	27.71	27.60	27.24		
	28.59	29.68	29.60	29.34	29.14	29.40	28.61	27.40	
	29.12	30.58	31.38	30.05	30.46	30.79	28.92	29.03	
29.60	29.79	30.62	31.50	31.71	31.10	31.26	30.64	29.62	27.68
29.89	30.45	30.31	30.41	31.96	32.04	31.14	30.52	29.45	28.04
	29.45	30.58	31.47	30.32	30.72	31.78	30.12	28.68	
	29.30	29.71	29.79	29.94	29.96	30.16	29.89	29.10	
		28.46	29.49	28.31	28.92	28.86	28.51		
				27.79	27.80				

Fovea = 32.79

Supplementary Figure 1: Mean, age-corrected, contrast sensitivity for all test locations in the 10-2 paradigm with five different stimulus sizes for all normal participants (corrected to a 50-year-old age equivalent). The lower 5th percentile distribution limit for GIII is also provided for analysis against disease cohorts. All values are in dB.

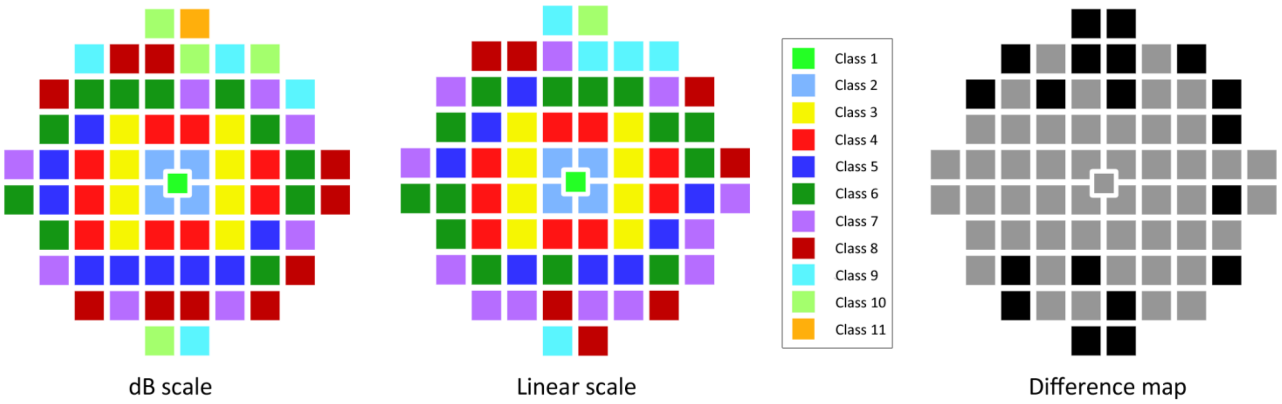

Supplementary Figure 2: Hierarchical cluster analysis of sensitivity values across the 10-2 test grid measured using GIII in dB (logarithmic scale), 1/Lambert (linear scale) and a difference map highlighting locations assigned to a different class between the two analyses. The theme maps generated from the respective scales showed an overall similar distribution of CSIs although there were some differences in the peripheral CSIs. This analysis suggests that the general concentric distribution of CSIs is maintained following conversion of sensitivities from logarithmic (dB) to linear (1/Lambert) scale.
